# Comparison of Morphological Characteristics and Determination of Different Patterns for Rubber Particles in Dandelion and Different Rubber Grass Varieties

**DOI:** 10.3390/plants9111561

**Published:** 2020-11-13

**Authors:** Boxuan Yuan, Guohua Ding, Junjun Ma, Lingling Wang, Li Yu, Xueyu Ruan, Xueyan Zhang, Wangfeng Zhang, Xuchu Wang, Quanliang Xie

**Affiliations:** 1 Key Laboratory of Xinjiang Phytomedicine Resource and Utilization of Ministry of Education, College of Life Sciences, Shihezi University, Shihezi 832003, China; 15830503299@163.com (B.Y.); yulixjnu@163.com (L.Y.); 2 Key Laboratory for Ecology of Tropical Islands, Ministry of Education, College of Life Sciences, Hainan Normal University, Haikou 571158, China; dingguohuasw@163.com (G.D.); majunjun_edu@163.com (J.M.); wll_198927@126.com (L.W.); ruanxueyu18@126.com (X.R.); zhangxueyan_caas@126.com (X.Z.); 3 The Key Laboratory of Oasis Eco-Agriculture, Agricultural College, Xinjiang Production and Construction Corps, Shihezi University, Shihezi 832003, China; zhwf_agr@shzu.edu.cn

**Keywords:** morphological characteristics, natural rubber, rubber grass, rubber particle, Russian dandelion, *Taraxacum kok-saghyz*

## Abstract

Russian dandelion *Taraxacum kok-saghyz* (TKS) is one promising alternative crop for natural rubber production. However, it is easily confused with other dandelions. In this study, we performed a systematical comparison of the morphological characteristics for different TKS varieties and common dandelion *Taraxacum officinale* (TO). Our results demonstrated that several obvious differences in morphology can be found between TKS and TO. TO leaf is a pinnate shape, its margin is heavily jagged and its base is cuneate, but TKS leaf is more cuneate and its leaf margin is nearly smooth and round. There are obvious differences for the outer bracts of TO and TKS flower buds. TKS bracts are oblanceolate, apex obtuse, margin smooth and sinuate, and its outer layer of flower buds and faceplate involucre sepal is buckled inward to form a certain angle. TKS is self-incompatible, and its seeds are spindle-shaped achene and show upright plumpness. A large amount of laticifer cells and rubber particles can be detected from many TKS tissues, and dry roots of TKS contain high contents of natural rubber. Laticifer cells and rubber particles can only be examined in the vein, stem, and roots of TKS. Our statical results also revealed that the numbers of laticifer cells and rubber particles have a positive relationship with the rubber content in TKS roots. These morphological features can help us to easily distinguish TKS from common dandelion and approximately estimate the rubber content in the roots of different TKS varieties for TKS breeding in future.

## 1. Introduction

Dandelion is a weedy perennial herb of the genus *Taraxacum* of the family Asteraceae, and it is native to Eurasia but is widely distributed in the warmer, temperate zones of the northern hemisphere [1]. This genus is found to include more than 2800 species, which are divided into 60 classification sections [2]. In China, one of the most familiar species of dandelion is *Taraxacum officinale* (TO) [3]. Among these dandelion species, the Russian dandelion *Taraxacum kok-saghyz* Rodin (TKS) is the only member that contains high-quality natural rubber, with an average molecular weight of 2180 kDa in its root latex, which is very similar to that obtained from the latex of *Hevea brasiliensis* [4]. TKS was first found in the border area of Kazakhstan and China and it grows widely in high valleys of the Tian Shan Mountains [5]. In its mature roots, approximately 5% natural rubber and 25–40% inulin have been detected on a dry weight basis in wild germplasm. Therefore, TKS has been considered as a potential alternative rubber crop in temperate regions [4,6].

Natural rubber (cis-1,4-polyisoprene) is widely used as an industrial and strategic raw material in nearly 50,000 products [7,8]. There are than more 12,500 latex-producing plants [9]; among them, approximately 2500 higher plant species can biosynthesize natural rubber [10,11,12], and these rubber-producing plants are distributed in at least eight families, indicating that natural rubber occurrence is polyphyletic in the plant kingdom [7]. However, until today, the Para rubber tree (*Hevea brasiliensis* Muell. Arg.) has been nearly the only economic crop utilized as the commercial source to produce natural rubber [13,14]. However, rubber production ability in *H. brasiliensis* has almost reached the limit due to its limited genetic variability [15,16], threats of fatal fungal plant diseases [17], the strict tropical climate requirements for its planting areas [15], and the increasing labor cost [16,17]. With the increase in demand for natural rubber in various countries, it is critical to find an alternative source and a model plant for natural rubber production [11].

Recently, the genome of TKS has been completed and many key genes in the mevalonate (MVA) pathway for regulation of natural rubber biosynthesis (NRB) have been annotated [3]. The results showed that TKS is a diploid (2*n* = 16) with a relatively simple genome with a length of 1.29 Gb, which contains 46,731 predicted protein-coding genes [3]. TKS roots contains high-molecular-weight rubber molecules and their characteristics are comparable to those in the rubber latex of *H. brasiliensis* [18,19]. As an alternative rubber crop, TKS roots contain a high rubber content, ranging from 3% to 28% of their total dry weight [17], and they are also a new source of inulin (a linear β-(2-1)-linked fructan), which is another abundant dandelion metabolite stored in parenchymal root cell vacuoles near the phloem, adjacent to apoplastically separated laticifers, and is an important material for the food industry and bioethanol production [14,20,21]. In addition, TKS can widely grow in cold and temperate areas with a relatively short life cycle [17], and it is easy to harvest and perform genetic transformation and, thus, can be used as an ideal model plant for studying the gene functions for natural rubber biosynthesis [14,20,21].

In rubber-producing plants, a specific organelle, named the rubber particle, has been evolved to synthesize and store natural rubber in its laticifer cells [22]. It is a specialized spherical organelle that is surrounded by a lipid monolayer and membrane-bound proteins [7]. Morphological observation results demonstrated that the size of different rubber particles in various rubber-producing plants is different, and the number of rubber particles also varies among different plant species. It was reported that the average diameter of rubber particles in *H. brasiliensis* is approximately 1 μm [23], but our recent transmission electron microscopy (TEM) and scanning electron microscopy (SEM) results revealed that the diameter of the examined 2600 rubber particles in the *Hevea* laticifer cells ranged from 50 to 2500 nm, and most rubber particles have a diameter ranged from 100 to 300 nm, whereas the 871 large rubber particles account for 33.5% (larger than 400 nm) of the examined 2600 rubber particles [8]. In *Ficus elastica* and *Ficus* spp., the estimated mean diameter of rubber particles ranged from 1.6 to 6.0 μm [23,24], which seems larger than those in the *Hevea* rubber tree. However, many more numbers of smaller rubber particles (less than 0.35 μm) are found in the laticifer cells of mature TKS roots [10], and the specific relationship between the size of rubber particles and the yield of natural rubber production has not been systematically studied.

In this study, the morphological characteristics of leaves, seeds and gelatinous roots in common dandelion and TKS were compared and different patterns for rubber particles in different tissues of rubber grass varieties and *H. brasiliensis* were systematic determined. Our morphological results may help potential researchers to distinguish the common dandelion and the TKS in the field and select the valuable germplasm with high natural rubber content by comparing the patterns of their rubber particles, thus accelerating the breeding process for TKS as a new commercial crop for natural rubber production in future.

## 2. Results

### 2.1. Comparison of Morphological Characteristics of Common Dandelion, T. officinale, and Russian Dandelion, T. koksaghyz

**Figure 1:**
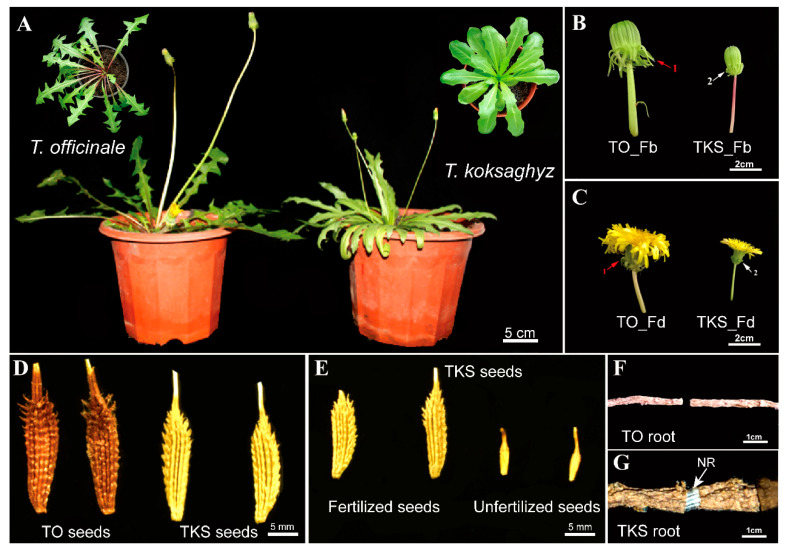
Morphological analysis of *Taraxacum officinale* (TO) and *T. koksaghyz* (TKS). Mature TKS (**right**) and TO (**left**) grown on semi-solid nutrient are presented (**A**). The flower buds (**B**), flowers (**C**), mature seeds (**D**), unfertilized seeds (**E**), mature roots (**F**), and root breaks (**G**) are highlighted to demonstrate the differences between the TKS and TO species.

Both *T. officinale* and *T. koksaghyz* belong to dandelion, and they look like the same species at first glance (Figure 1A). The two species are mainly in the form of a rosette and commonly found in an ecotonal habitat in floodplain meadows, saline-alkali meadows, dry stands of wet, saline bunchgrass meadows, and farmland canals. For seed propagation, plants were cultivated from a mixed set of seeds of these accessions in the greenhouse, *Taraxacum* plants were found which closely fitted the original descriptions of *T. koksaghyz*. However, there are several different patterns in morphology between them. The leaves of TKS and common dandelion are rosette-shaped. The leaf of TO is a pinnate shape, but the leaf of TKS is more cuneate. The TO leaf margin is heavily jagged and its base is cuneate. The blade tip is sharp and the base of the main vein is reddish-brown, in the form of lateral parallel veins. TKS leaf shape is also cuneate, the leaf margin is nearly smooth and round, the leaf base is also cuneate, and the blade tip is half a circle. The base of the main vein is white or red, also in the form of lateral parallel veins (Figure 1A). The flowers of *Taraxacum* are capitulum, but there are obvious differences in morphology between the outer bract of TO and TKS flower buds (Figure 1B). The TO flower bud outer bract is outward stretching, and the outer bracts of TKS flower bud will form an angle inward (Figure 1C). The dandelion seeds were observed under a light microscope and the spindle morphology belonged to achene. TO and TKS seeds are dark brown and dark green, respectively (Figure 1D). The morphology of the seed surface is arranged in a profile of ridges and minor groove, and a barb appears in the second half of the ridges. The morphological characteristics of seeds are conducive to rapid penetration into the soil after landing. Due to TKS having self-incompatibility, we also observed the morphological differences of fertilized and unfertilized seeds (Figure 1E). The *T. koksaghyz* fertilized seed surface has a clear contour, large individual seeds and plumpness. The unfertilized seed surface has a fuzzy contour and is slim and degenerative. Drying their roots and breaking the roots, we found that there is no colloidal wire drawing at the TO root break (Figure 1F), and there was a continuous white colloidal wire drawing at the TKS dry root break (Figure 1G).

### 2.2. Determination and Statistical Analysis of Laticifer Cells in Different Tissues of T. koksaghyz

**Figure 2:**
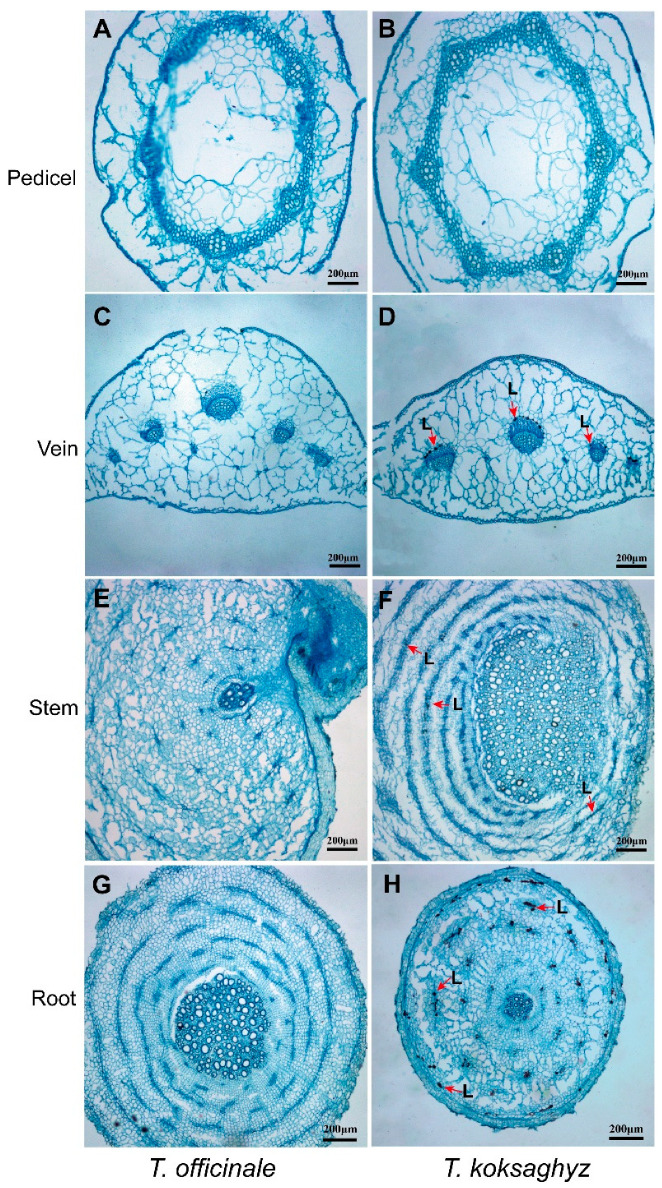
Morphological comparison of laticifer cells in different tissues of *T. officinale* and *T. kok-saghyz*. The laticifer cells containing brown rubber latex strands can be examined under a light microscope in the cross-cutting sections of the vein (**D**), stem (**F**), and main roots (**H**) in TKS, but no laticifers were observed in TKS pedicel (**B**) and in all the detected tissues in *T. officinale* (**A**,**C**,**E**,**G**).

**Figure 3:**
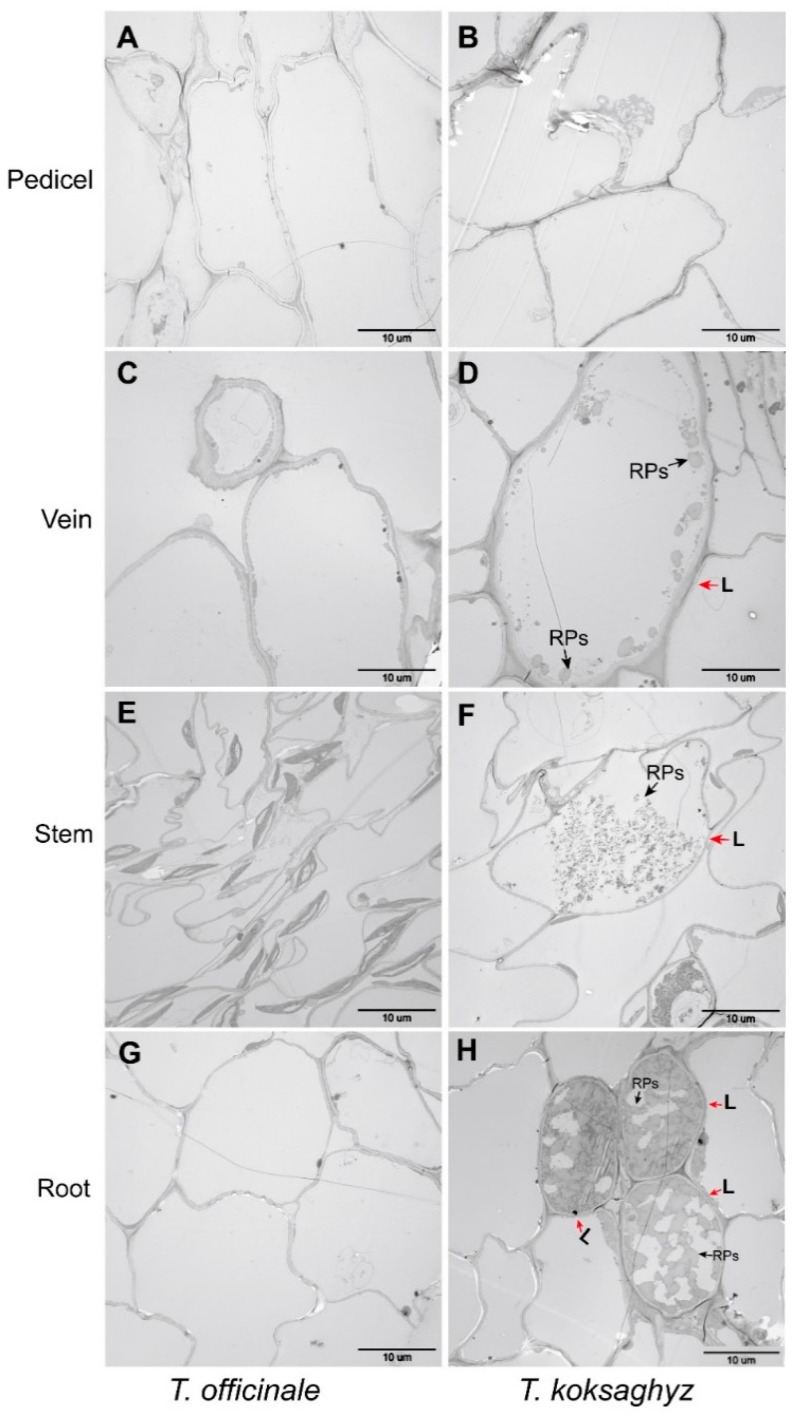
Accumulation patterns of rubber particles and laticifer cells in different tissues of *T. officinale* and *T. kok-saghyz*. Typical ultrastructural patterns of rubber particles and laticifers (L) in different TKS tissues were examined under a TEM, which are marked with black and red arrows, respectively. Many spherical and pear-like rubber particles can be detected (**D**,**F**,**H**) in TKS (Vein, Stem, Root), but both rubber particles and laticifer cells cannot be found in the pedicel tissue of TKS (**B**) and all the detected tissues in *T. officinale* under a TEM (**A**,**C**,**E**,**G**).

A light microscope was used to examine the patterns of laticifer cells in different TKS tissues, and the corresponding tissues in the common dandelion *T. officinale* were also used as control samples. Our morphological observation results revealed that the concentric rings of laticifer cells as specialized tubular vessels in different tissues can be detected in most tissues of TKS but cannot be observed in all of the examined TO tissues (Figure 2). Cross-sections in the vein (Figure 2D), stem (Figure 2F), and main roots (Figure 2H) of TKS show brown-stained latex strands in the concentric rings of latex granules, which are known as laticifer cells. It is noteworthy that laticifer cells cannot be detected from the pedicel tissues of both TO (Figure 2B) and TKS (Figure 2B). A substantial accumulation of latex reserves can be observed in the lignified part of the vein and stem tissues in TKS, but laticifer cells have not been detected in vein and stem tissues in common dandelion. In the cross-section of the main roots in TKS, large amounts of brown rubber latex granules, known as the specific cytoplasm in laticifer cells, can be clearly detected in the areas of endodermis and cortex in phloem tissues from both the transect and transverse slices of the main roots (Figure 2H). Our results showed that all the laticifer cells may originate from the initial cells of vascular cambium to secondary laticifers in parallel rings (Figure 2D,F,H). Ultrastructural observation results also supported that laticifer cells can only be examined in the vein, stem, and roots from TKS, but cannot be detected from these tissues in TO (Figure 3).

The total number of laticifer cells and the natural rubber content in different tissues of the six-month-old (6M) TKS-S variety were examined and the results are provided as the supplementary data (Table S1). Our statistical results demonstrated that approximately 1891 ± 143 cells can be examined from the cross-section of the main roots from these mature TKS, and among them, 137 ± 32 (*n* = 10) laticifer cells can be observed to accumulate large amounts of brown rubber granules, which means about 7.3% of the detected cells in the main roots are termed as laticifer cells. We further detected the rubber content and found 2.74% ± 0.75% (*n* = 10) of dry weight rubber content can be checked from these roots. Compared to roots, less total cells (411 ± 21), fewer numbers (40 ± 13, *n* = 10) of laticifer cells, and much lower rubber content (0.56%) were observed in the vein tissues, but a higher percent (9.67%) of laticifer cells was examined from the veins of the mature leaves in TKS. More than three thousand (3569 ± 225) cells were detected from the cross-section of TKS stems, and 8.71% of these cells were determined as laticifer cells, and the natural rubber content is similar with that in the roots, which is about 2.75% in dry weight matter. It is noteworthy that there are no laticifer cells in the checked pedicels (Figure 3B), and the corresponding rubber content is zero in the pedicel tissues (Table S1).

### 2.3. Morphological Analysis of Laticifer Cells and Rubber Particles in the Nine-Month-Old Roots of T. kok-saghyz from Different Varieties and the Rubber Tree H. brasiliensis

**Figure 4:**
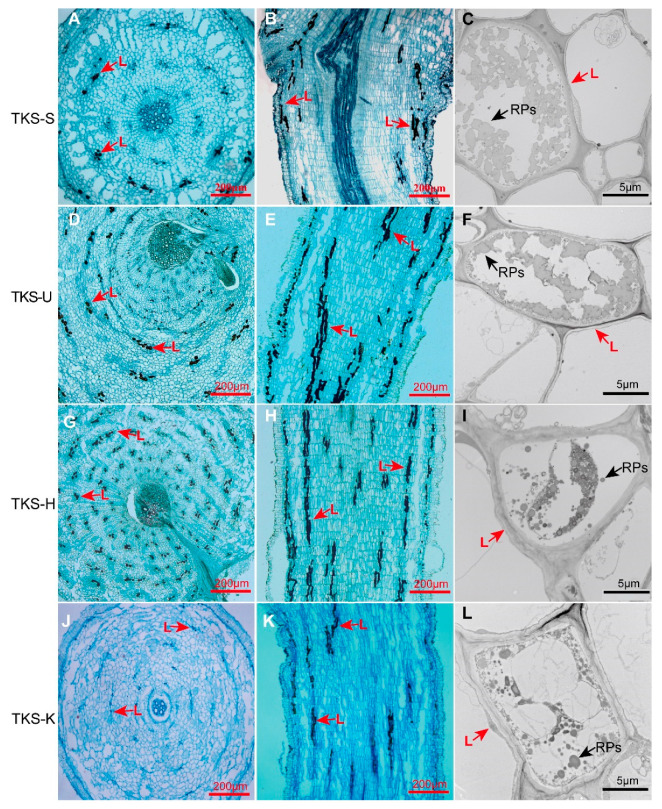
Comparison of rubber particles and laticifer cells in the old roots of different *T. kok-saghyz* varieties. Distribution of laticifer cells in the cross-cutting (**A**,**D**,**G**,**J**) and longitudinal sections (**B**,**E**,**H**,**K**) in old roots of four TKS varieties were highlighted, and rubber particles in their laticifer cells were further examined under a TEM (**C**,**F**,**I**,**L**) in TKS-S (**A**–**C**), TKS-U (**D**–**F**), TKS-H (**G**–**I**), and TKS-K (**J**–**L**).

Furthermore, we observed the distribution of laticifer cells in the nine-month-old (9M) roots of different rubber grass varieties (TKS-S, TKS-U, TKS-H and TKS-K), which contain different natural rubber content in their roots (Figure 4). Laticifer cells in both the cross-cutting and straight-cutting sections were examined under a light microscope and the patterns of rubber particles (RPs) in these roots were further checked under a TEM (Figure 4). Under a light microscope, these laticifer cells were imbued with brown rubber bast and they were distributed around or in the xylem, endodermis, phloem, endodermis, and epidermis tissues. The largest number of laticifer cells was observed in TKS-H roots, followed by TKS-U and TKS-S, and the least laticifers were found in both the cross-cutting and straight-cutting sections from the nine-month-old (9M) roots of TKS-K variety. From the cross-cutting sections of 9M roots of TKS-H variety, approximately 9170 cells were observed in each root, and among these cells, 1140 ± 93 (*n* = 101) were determined as laticifer cells, which accounts for 15.7% ± 1.5% of all the detected cells. From TKS-U, 10,787 ± 1395 cells were detected in each root, and about 10.1% of them were laticifer cells. Approximately 605 laticifer cells were determined from 8102 cells in the roots of TKS-S, which accounts for 7.5% in all checked cells. The lowest percent of laticifer cells was detected from TKS-K, and only 123 ± 34 out of 8384 ± 424 (*n* = 100) laticifer cells had been examined in the 9M roots (Table S2).

**Figure 5:**
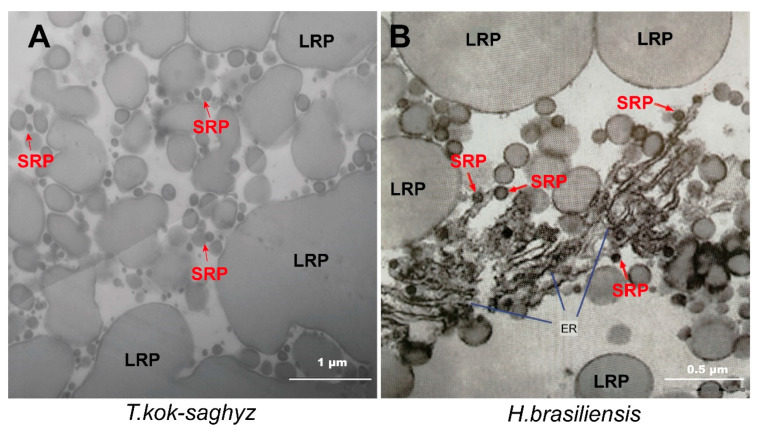
Morphological patterns of rubber particles in laticifer cells of the rubber grass *T. kok-saghyz* and rubber tree *H. brasiliensis*. Many pear-like and spherical large rubber particles (LRP, black color) and small rubber particles (SRP, red color) can be detected under a TEM, and typical ultrastructural patterns of these rubber particles in *T. kok-saghyz* (**A**) and *H. brasiliensis* (**B**) are highlighted.

Under a TEM, large amounts of rubber particles could be found in the four kinds of TKS variety, and these rubber particles appeared approximately round, ovoid-ellipsoid, spherical, or pear-shaped, with diameters ranging from 5 to 5000 nm (Figure 4). The ultrastructural patterns of rubber particles in laticifer cells from TKS and the rubber tree *H. brasiliensis* were further compared, and many more smaller rubber particles could be examined in TKS laticifers than those in TKS roots (Figure 5). All of these rubber particles are surrounded by a monolayer membrane (Figure 5), in which many enzymes related to natural rubber biosynthesis (NRB) are found to anchor or combine with the membrane. Our ultrastructural observation in the rubber tree revealed that very small rubber particles may originate from endoplasmic reticulum in the cytoplasm of laticifer cells (Figure 5B).

### 2.4. Comparison of the Accumulation Pattern of Different Rubber Particles and the Natural Rubber Content in Different Rubber-Producing Plants

In the laticifer cells, two distinct kinds of rubber particles were observed from the rubber tree *H. brasiliensis* (Figure 5B) and rubber grass *T. kok-saghyz* (Figure 5A). The diameter in the examined rubber particles ranged from 5 to 5000 nm. Rubber particles with a diameter less than 200 nm are traditionally called small rubber particles (SRPs), while the others with a diameter larger than 400 nm are fewer in number and they are termed large rubber particles (LRPs). We determined the diameter of 1314 rubber particles with an average diameter 374 nm. Among them, 564 rubber particles (SRPs, about 42.92%) had a diameter smaller than 200 nm and 472 were larger than 400 nm (LRPs, about 35.93%). In this study, the largest rubber particle in the rubber tree was about 2200 nm (Table S3).

**Figure 6:**
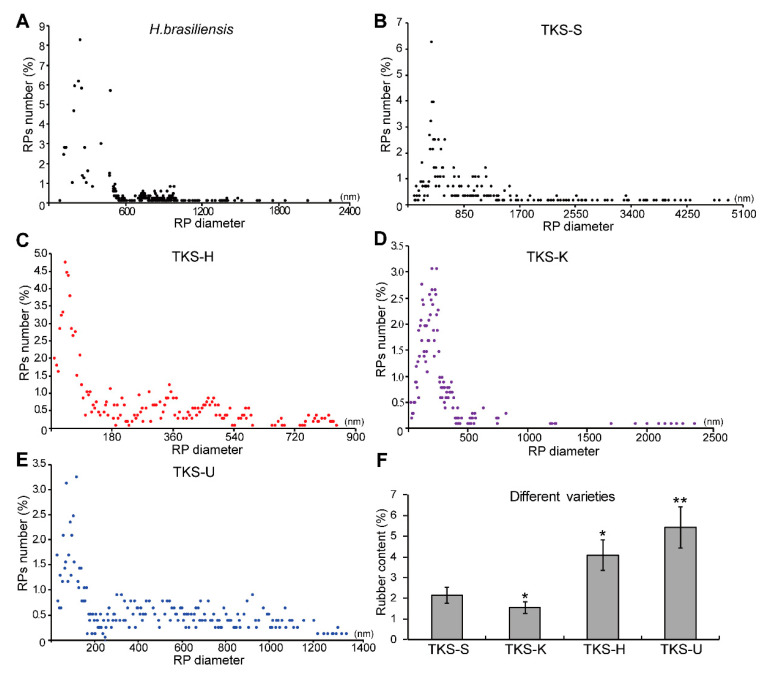
Statical analysis of rubber particle accumulation patterns and determination of rubber content in different rubber-producing plants. The percentages of different sized rubber particles in *H. brasiliensis* (**A**) and different *T. kok-saghyz* varieties (**B**–**E**) were calculated based on their diameters. The content of natural rubber in the four TKS varieties was further determined, and the highest rubber content was obtained from the old roots of TKS-U variety (**F**).

The sizes of rubber particles in different TKS varieties are different from each other (Figure 6B–E). The rubber content in the mature roots of these TKS varieties is also different. The rubber particles of TKS-S and TKS-U are irregularly spherical and there is a fusion phenomenon between the rubber particles membrane. However, the rubber particles in TKS-H and TKS-K have a clear background and these rubber particles are spherical or globular (Figure 4).

The highest rubber content was examined in TKS-U, which contained 5.43% ± 1.12% rubber in their 9M roots. Correspondingly, more LRPs could be detected in their roots. For the 768 rubber particles from the old roots of TKS-U, 327 SRPs and 324 SRPs (about 42.23%) were determined. The average diameter for rubber particles in TKS-U was 398 nm, and the largest one was 1351 nm (Figure 6E). For TKS-H, about 4.17% natural rubber could be detected from their mature roots, and the average diameter for rubber particles was 220 nm. Among the 1051 examined rubber particles from the old roots of TKS-H, 614 rubber particles (accounting for 58.43%) had a diameter smaller than 200 nm, and 232 LRPs (about 22.12%) were determined. Among these LRPs, the largest one was only with a diameter of 844 nm, which is much smaller than that in other TKS varieties. Compared to TKS-U and TKS-H, TKS-S contain less natural rubber (about 2.16% ± 0.41%) in their roots (Figure 6F). In TKS-S, only 21 out of 557 rubber particles had a diameter smaller than 200 nm, which accounts for 3.83%. A total of 348 LRPs were detected from these rubber particles, which accounts for 62.48% of all the examined rubber particles. Among these LRPs, the diameter of the largest rubber particle was 5060 nm, which is also the largest one in all the detected rubber particles from both the rubber tree and rubber grass. The lowest rubber content was obtained from TKS-K, which was only about 1.57% ± 0.16% in the detected roots. In TKS-K, 53 out of the 1013 rubber particles had a diameter larger than 400 nm, and these LRPs account for only 5.23%. More than one half of the members in these TKS-K rubber particles had a diameter less than 200 nm, and 15.39% of these SRPs were smaller than 100 nm (Table S3). These calculated results, as well as the observations in the TEM images, revealed that the proportion of LRPs has a positive relationship with rubber content, and a large number of LRPs indicates a higher rubber content in the roots of TKS varieties.

## 3. Discussion

*T. kok-saghyz* constitutes a promising source for valuable raw materials, such as natural rubber, insulin, bio-ethanol, and other secondary metabolites. However, the large genetic diversity of TKS leads to an immense phenotypic variance, meaning that the content of metabolites can differ drastically among different individuals [25]. Thus, a comprehensive understanding of the fundamental developmental and metabolic processes of the plant root and its laticifers are required to successfully establish *T. kok-saghyz* as a suitable crop plant. In field, the common dandelion *T. officinale* is often confused with the Russian dandelion *T. kok-saghyz.* In history, a close species named *T. brevicorniculatum*, which commonly co-occurs with wild populations of *T. kok-saghyz*, has been used as TKS for a long time [19]. The published data showed that seeds collected from *T. kok-saghyz* at the end of the 1960s have been contaminated with other apomictic *Taraxacum* species. Herbarium analysis revealed that *T. kok-saghyz* seed batches in the past were sometimes contaminated with *T. brevicorniculatum*, and the *T. kok-saghyz* seeds collected from field were usually contaminated with at least three different types of rogues; they are Russian *Taraxacum* species, American *T. officinale*, and a Russian *Taraxacum* species with red achenes [19]. These results revealed that seed contamination is a serious problem in TKS cultivation, and it is necessary to establish an effective evaluation system to eliminate the confusion of TKS and *Taraxacum* species.

In order to re-find wild *T. kok-saghyz*, an expedition was organized in June 2008 to reexplore the river valleys in the mountains with altitudes between 1700 and 2000 m in eastern Kazakhstan; many *Taraxacum* plants were found and some of them closely fitted the original descriptions of *T. kok-saghyz* in the 1430s [26]. These *Taraxacum* species commonly grow in an ecotonal habitat between wet saline meadows and dry stands of bunchgrass, and they have horned bracts, but with horns shorter than those of *T. kok-saghyz* [19]. Our morphological observation results also showed that Russian dandelions *T. kok-saghyz* are distinguished from TO, especially in their leaf shape and horned bracts (Figure 1).

Based on a historical section technique, Warmke found that *T. kok-saghyz* is a basic diploid, and sixteen chromosomes (2*n*) were detected in its cells [27]. An early rapid histological staining result demonstrated the cross-section of TKS roots contain darkly-stained latex strands in the concentric rings of latex tulles [28], which is consistent with our morphological observation in this study (Figure 2), by producing a new developed mercury-bromophenol blue-staining method for showing the proteins in rubber latex [29].

We also noticed that in the published literature, the relationship among the numbers of lactiferous cells and rubber particles and the rubber content are not well compared. It was reported that the age of the plants appeared not to affect particle size [10]. For rubber particles, more than 50% of them ranged from 250 to 400 nm in TKS, and the average size of the rubber particles was 320 nm [10]. These results have several differences with our observation of different TKS varieties (Figure 6; Table S3), and many more small rubber particles can be examined in our TKS roots, which may be produced by the different sample preparation methods for TEM. We performed an in situ method by fixing the tissue slices directly on 4% glutaraldehyde solution, but Schmidt used an in vitro way of observe rubber particles by TEM. They collected the rubber particles by the washing method [10], and many very small rubber particles may lose into the washed solutions [8]. Our results demonstrated that different TKS varieties have different rubber particle patterns, and more LRPs can be detected in the roots of high-rubber-content TKS varieties. Our results also indicated that the number of laticifer cells is positively correlated with rubber content (Figure 6).

Rubber particles are the main organelles of natural rubber biosynthesis (NRB) [30], and many crucial proteins involved in the NRB process have recently been identified from rubber particles by mass spectrometry [7,31,32]. These proteins include Cis-prenyltransferase (CPT), rubber elongation factor (REF), small rubber particle protein (SRPP), HRT1-REF bridging protein, a Nogo-B receptor (HRBP), isopentenyl pyrophosphate (IPP), etc. NRB is a typical isoprenoid metabolic process [33,34], and our recently published results revealed that both the Mevalonate acid and Methylerythritol phosphate pathways are important for NRB in mature TKS roots [12]. Our proteomics results also revealed that SRPs might play more important roles than LRPs for NRB in *H. brasiliensis* [8]. Laticifer cells were considered to originate from a series of cells united by dissolution of intervening walls [35]. It was reported that in TKS seedlings, as early as 10 days after germination, small rubber particles start to form in the laticifer cells, and these rubber particles have similar morphological patterns to those in other rubber-producing plants, such as *H. brasiliensis* and *Parthenium argentatum* [35].

As an herbaceous plant, *T. kok-saghyz* can grow broadly in temperate regions [14]. However, it cannot be planted as a commercial economic crop yet due to its limited yield [36]. *T. kok-saghyz* is largely undomesticated and has several inherent issues that need to be addressed, such as its self-incompatibility and high degree of heterozygosity, poor competitiveness with weeds, slow growth rate, need for steady moisture content during germination, and meaningful rubber yield only obtained from the mature roots [37]. Recent years have seen a renewed interest in the possible development of TKS as a rubber crop [36], and burgeoning TKS research has led to field-scale trials to evaluate the rubber performance in its roots [4,6]. Therefore, it is essential to distinguish TKS from TO before TKS can become a commercially viable and competitive rubber crop that needs conventional and molecular breeding efforts to further improve its agronomic fitness and rubber yield [6,14,20]. Furthermore, established farming practices are some of the key bottlenecks that still face TKS development as a new industrial rubber crop [6].

Based on our morphological comparison of TKS and TO by light microscopy and TEM methods, we considered that there are five special characteristics for the morphology of *T. kok-saghyz*. Firstly, the TKS leaves are oblanceolate, apex obtuse, margin smooth, and sinuate. Secondly, the outer layer of flower buds and faceplate involucre sepal buckle inward to form a certain angle, which is one of the most obvious distinguishing traits. Thirdly, TKS is self-incompatible, the seeds have two forms, with one of spindle-shaped achene and upright plumpness, and the incomplete developed seeds are smaller than the fertilized ones. Fourthly, TKS dry roots contain a high content of natural rubber, and when they are broken and pulled to both ends, many gelatinous strands will be observed (Figure 1). Fifthly, many laticifer cells can be detected from different TKS tissues, and large amounts of rubber particles can be examined in these laticifer cells; the numbers of laticifer cells and rubber particles have a positive relationship with the rubber content in TKS roots, whereas no laticifer cells and rubber particles can be found in common dandelions *T. officinale* (Figure 2, Figure 3, Figure 4 and Figure 5). These morphological features can help us to easily distinguish TKS from common dandelions and approximately estimate the rubber content in the roots of different TKS varieties in the future.

## 4. Materials and Methods

### 4.1. Plant Materials and Growth Conditions

The mature achenes of seeds from *T. officinale* and *T. kok-saghyz* were collected from the Tekes River Basin from Xinjiang Province in China. Ten-year-old regularly-tapped rubber trees (*H. brasiliensis* Mull. Arg., clone RY 7-33-97) were grown in the Experimental Farm of the Chinese Academy of Tropical Agricultural Sciences (CATAS) in Danzhou city, Hainan province, P. R. China. Ten of the regularly-tapped trees were selected to observe the plug formation and accumulation at the severed laticifers. Four varieties of *T. kok-saghyz* (named TKS-S, TKS-U, TKS-H, and TKS-K) with different natural rubber contents in their roots and one common dandelion, TO, were used in this study. Achenes were previously removed from the pappus. The seeds were treated with distilled water for 5 min. After rinsing with distilled water, TKS and TO seeds were surface-sterilized by immersion in a solution of 10% (*w*/*v*) sodium hypochlorite for 12 min, followed by six washes with sterile water for 3 min each. We stratified sterilized seeds at 4 °C in the dark for 2 days before plating. After germination, seedlings were transplanted into the pots in a greenhouse at Hainan Normal University (Hainan, China). After 1 month of acclimatization, plants were transferred to 1-L plastic pots containing vermiculite and nutritive soil with a ratio of 3:5, and a half strength of Hoagland solution was irrigated with 50% relative humidity at 18 °C in the dark and at 23 °C in the light. After growing for 3 months, at the flowering stage, the different tissues from plants were collected, rinsed with distilled water, and blotted dry with filter paper, and the middle parts of tissues were dissected into approximately 0.5–1.0-cm thick slices and then frozen in liquid nitrogen for further study.

### 4.2. Morphological Observation of Laticifer Cells in Different Tissues

For histochemical staining, approximately 0.5-cm long sections from the middle parts of pedicels, leaves, stems, and main roots from TKS and TO were fixed in 4% glutaraldehyde (0.1 M phosphate buffer, pH 7.5) for 48 h at room temperature. These fixed samples were then dehydrated through a graded series of ethanol and embedded in paraffin as described [29]. The slices in these sections (12-μm thickness and area of 5 mm × 5 mm) were generated by a microtome (Leica Microsystems, Bannockburn IL, Germany) and then stained with mercury–bromophenol blue, which is effective in showing the latex proteins in laticifers [34]. Finally, these stained slices were examined under a Leica DMLN light microscope (Leica, Wetzlar, Germany). TKS laticifer cells in the root sections could be recognized due to iodine-bromine treatment of the rubber in the laticifers, which become a deep brown or dark color. Another part of these samples was dehydrated in a range of acetone (30%, 50%, 70%, 90%, for 1 h, once, and then, in pure acetone for 2 h, twice). Then, these slices were permeated with acetone and Epon 812 embedding agent and pure Epon 812 embedding agent permeates overnight, as described [24]. After that, the samples were polymerized in the oven, flattened, and cut into semi-thin slices. Under an optical microscope, the laticifer cells could be recognized by tracing rubber inclusions, which are brown-stained by iodine-bromine.

### 4.3. Morphological Analysis of Large and Small Rubber Particles

TEM analysis was performed to observe the rubber particles, as described [29,38]. Ultra-thin slices were double stained with uranium acetate and lead citrate. A transmission electron microscope (TEM, Hitachi JEM-1230, JEOL, Tokyo, Japan) was used to examine these stained slices to examine the ultrastructural patterns of laticifer cells and rubber particles in TKS and *H. brasiliensis*.

### 4.4. Determination of Natural Rubber Content

An infrared spectroscope was used to measure the content of the natural rubber in the TKS roots. At first, petroleum ether extracts were obtained after water and acetone extraction of 100 mg dry tissues. Each sample was extracted 3 times by acetone ether in order to completely remove the water and pigment extractable materials. After centrifugation, the supernatant was discarded and the acetone solution was allowed to evaporate at 55 °C in a drying oven for 35 min. Then, 3 mL of petroleum ether was used to extract the dried materials 3 times, and the extracts were combined to determine the rubber content by using Fourier transform infrared (FTIR) spectroscopy (Thermo Scientific, Nicolet™ 6700 FT-IR spectrometer, Waltham, MA, USA) as described [39,40].

### 4.5. Statistical Analysis

For all the generated data, at least three biological replicates were performed for each sample. The statistical significance (*p* values) in mean values was determined with an unpaired two-tailed Student’s *t*-test (Microsoft Excel). Then, a one-way analysis of variance (ANOVA) multiple range test was performed at a 5% significance level using the Origin software (version 9.0). The statistical results were reported as the means ± SD. The significance level of *p* < 0.05 was considered to indicate statistical significance.

## Supplementary Materials

The following are available online at https://www.mdpi.com/2223-7747/9/11/1561/s1, Supplementary Table S1: Number of laticifer cells and rubber content in different tissues of *Taraxacum kok-saghyz* (TKS-S) variety; Supplementary Table S2: Number of laticifer cells in mature roots of different TKS varieties, Supplementary Table S3: Number of rubber particles in the rubber tree and different TKS varieties.
